# An Educational Program Based on the Successful Aging Approach on Health-Promoting Behaviors in the Elderly: A Clinical Trial Study

**DOI:** 10.5812/ircmj.16314

**Published:** 2014-04-05

**Authors:** Fatemeh Estebsari, Mohammad Hossein Taghdisi, Abbas Rahimi Foroushani, Hasan Eftekhar Ardebili, Davoud Shojaeizadeh

**Affiliations:** 1 Department of Health Education and Promotion, School of Public Health, Iran University of Medical Sciences, Tehran, IR Iran; 2 Department of Epidemiology and Biostatistics, School of Public Health, Tehran University of Medical Sciences, Tehran, IR Iran; 3 Department of Health Education and Promotion, School of Public Health, Tehran University of Medical Sciences, Tehran, IR Iran

**Keywords:** Community Health Planning, Aging, Health Promoting Intervention

## Abstract

**Background:**

Many criteria of successful aging are directly connected with Health-Promoting Behaviors.

**Objectives:**

The current study aimed to evaluate the effect of an educational program based on the successful aging approach on health promoting behaviors in the elderly.

**Patients and Methods:**

This clinical trial study was conducted on 464 Iranian elderly people over 60 years who were admitted at Health Houses for 12 months. Participants were selected through a two-stage cluster sampling and were placed in the control and intervention groups (232 participants in each group). The data collection tools included: a demographic checklist, Palmore Facts on Aging Quiz and the second version of Health Promoting Lifestyle Profile. The intervention was designed based on adult strategy education in five 45-minute sessions. The data obtained 3 months after the intervention were compared with the data obtained before the intervention. The data were analyzed using the descriptive and analytical tests such as paired T-test with SPSS version 20, at the statistical significant level 0.05.

**Results:**

The mean age of the participants in this study was 65.9 ± 3.6 (range 60-73). Results showed a statistically significant difference between the intervention and control group after the intervention in the mean scores of awareness of aging facts and score of health promoting behaviors.

**Conclusions:**

Focusing on successful aging and adopting HPBs can prevent and decrease aging problems which in turn decreases the financial burden and related costs. This is especially important for the policy and decision makers of the health systems.

## 1. Background

The population of the world is aging. It is anticipated that in 2050, the population of the elderly is much more than that of the children under five , reaching two billions 50 years from now (1). The aging pace in Iran and other developing countries is more than that of the developed countries. More than half of the world population (59%) is living in developing countries which is estimated to increase up to 71% in 2030 (2). Considering the rapid increase in the elderly population, their health and welfare needs are of great importance (3). Sedentary lifestyle, increased prevalence of mental and physical illnesses, insufficient and inefficient care, low income, and social isolation create a sense of loneliness and depression in the elderly, leading to the lower probability of Health-Promoting Behaviors (HPBs) (4). As a result of adopting an unhealthy lifestyle, the elderly people’s mental and physical health is in danger more than before. Health-Promoting Behaviors are one of the main health criteria and are directly connected to disease prevention (5). Based on the WHO researches, almost 60% of the quality of life and health depends on lifestyle and personal behaviors (6, 7). Also, 53% of the mortalities are attributed to lifestyle and health behaviors (2, 8, 9). Successful aging is defined as reaching one’s personal potentials and optimum physical, mental, and social capacities creating a sense of satisfaction in the elderly person (10). Successful aging is a combination of long life, health (absence of disabilities), and happiness that results in peace of mind until the end of life (11). Successful aging is affected by physical activity, nutrition (12), social interactions, active participation in social activities and interacting with people and society (12-14), general health status (15), disease status, cognitive physical and social functioning (16), age-associated changes degree (12), cognitive function, and good mental health (14). There is no agreement on the definitions of healthy aging and successful aging among researchers (12, 17) and they are mostly used interchangeably. Many criteria for successful aging and healthy aging are directly connected with HPBs (14, 18, 19). Several studies have expressed the positive relationship between a healthy lifestyle and successful aging (20, 21). The elderly who adopt HPBs will experience healthy aging (22). Sex, age, education level, marital status, housing status, lifestyle, social networks and support, income, behaviors related to healthy nutrition, physical activity, sleep, and entertainment are among the risk factors of successful aging (11). Considering the effect of personal lifestyle on health (2), adopting a healthy lifestyle is the most suitable way to decrease age related problems and have successful aging (18). It is necessary to design and develop educational programs for successful aging. Considering the importance and sensitivity of the subject as one of the challenges of the health systems, the present study aimed to evaluate the effect of an educational program based on the successful aging approach on HPBs in the elderly.

## 2. Objectives

The current study aimed to evaluate the effect of an educational program based on the successful aging approach on HPBs in the elderly.

## 3. Patients and Methods

### 3.1. Study Population and Sampling

After obtaining the necessary authorizations from the Ethics Committee of Tehran University of Medical Sciences (ID: 19230) and making the necessary coordination, the current parallel clinical trial was conducted on 464 elderly people over 60 years of age admitted at the Health Houses in 22 municipality diacritics in Tehran (the capital of Iran). The duration of the study was 12 months (between January 2012 and January 2013). The type of randomization was stratification. Participants were selected through a two-stage cluster sampling and were placed in the control and intervention groups (232 participants in each group). Based on the list of the 22 municipality districts in Tehran 44 Health Houses were randomly selected from 379 total Health Houses. Then names of these Health Houses were written on 44 cards. A card was selected from the 44 cards with each mixing. Therefore 22 cards were randomly selected. These 22 cards were assigned to the first group, and the remaining 22 cards were assigned to the second group.

**Figure 1.**
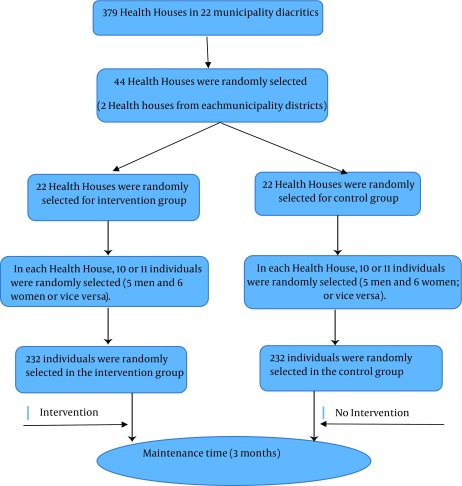
Flow Chart of RCT for This Study

Then, in each Health House, 10 or 11 individuals were randomly selected (five men and six women; or vice versa). A total number of 464 individuals (232 in the intervention and 232 in the control group) were selected (Figure 1). The two groups did not have any physical or geographical connections. The main purpose of this study was to examine the impact of education on the health promoting behaviors. This effect was assessed based on statistical diagram through the associations between education and other various factors. In these diagrams association was analyzed by correlation coefficient. So the sample size was determined based on the correlation coefficient matrix. In the current study the correlation coefficient 0.2 or more with Confidence level of 95% and the study power of 80% were significant. The sample size (193 elders) was determined by the following formula (Equation 1).

Equation 1. $$n=\frac{{\left(Z_{1-\frac{\alpha }{2}}-\frac{\alpha }{2}+Z_{1-\beta }\right)}^{2}}{Z_{0}^{2}}+3$$

### 3.2. The Sample Size Formula

To select the elders from Health Houses of the municipality diacritics, elders in each Health House were correlated with responses, according to the time and cost this sample size was multiplied in effect size (193 × 1.2 = 232) and the number of samples in each group (232) was determined. Researchers anticipated the possibility of losing some subjects from the start of sampling. There were 22 subjects that for various reasons were replaced. A total of 286 completed the research.

 To prevent bias, necessary correspondence was made with Elderly Centers of Tehran Municipality to make sure that no interventions similar to the current study were being conducted simultaneously. Inclusion criteria were age over 60 years, ability to speak Persian, lack of mental or psychological problems, and orientation to time and place. The elderly people who did not have the inclusion criteria were not included in the study.

### 3.2. Data Collection

The data collection tools included:

1. a demographic checklist: age, sex (male, female), marital status (single, married, widowed/divorced), education level (primary, secondary and higher), housing status (owner and rented), history of chronic diseases (yes, no), health insurance coverage (yes, no), living structure as who do you live with? (family, relatives, or alone). In this study family included spouse or children, and relative included sister, brother, friends and the others, and financial status (stable, unstable). Stable based on the pension, working or dependency to the other organizations, and unstable based on the dependency on children or without financial aid), perceived health status as how do you assess your current health? (Good, moderate or bad).
2. Palmore Facts on Aging Quiz (FAQ1) (23, 24). The questionnaire was used based on the previous studies showing that to increase the effect of educational interventions, part of the intervention should focus on awareness. As a standard tool, FAQ1 measures four educational objectives: learning, knowledge, awareness on the aging process, and false beliefs in this regard (3). The questionnaire has been developed and edited by researches in the field of aging in different countries (24) as well as Iran (25). The awareness tool included 25 questions answers as true (3 scores), false (2 scores) and I do not know (1 score). Cronbach's alpha was 0.81 showing that the internal consistency of the questionnaire was high and inter-class correlation was 0.98 showing acceptable reliability of the questionnaire in the retest of this present study. Awareness was classified into three levels: low (scores < 25), medium (scores between 25 and 50) and high (scores > 50).
3. The Health Promoting Behavior questionnaire: Using the second version of Health Promoting Lifestyle Profile (HPLP II), HPBs were measured using 52 questions in 6 subscales:
  1. Health responsibility.
  2. Physical activity,
  3. Nutrition,
  4. Spiritual growth,
  5. Interpersonal relations,
  6. Stress management.

The answers in the Likert scale ranged from “never” to “very frequently” and higher scores represented higher health-promoting behaviors. The tool has been translated into different languages (26) including Persian (9) and its validity and reliability has been assessed. In the present study, Cronbach's alpha was 0.84 showing that the questionnaire was acceptable for the study.

Trained interviewers completed the questionnaires through face-to-face interviews. To avoid bias and decrease its effects, all the questionnaires were completed simultaneously in the intervention and control groups. The questionnaires were anonymous and a coding system was used instead. After the completion of the questionnaires and preliminary data analysis, educational interventions were performed based on the results of the first stage under the supervision of the instructors in the field of health, health-promotion, and gerontology. Scientific resources were also used for the preparation of the course materials.

### 3.3. Educational Program Based on the Successful Aging

The successful aging based educational program was a Health-promotion educational intervention that included better identification of aging, knowledge of risk factors and threats of aging, physical activity, nutrition, social interactions, physical; mental; and spiritual health, and leisure time (27-30). The intervention was only performed in the intervention group. The intervention, which was designed based on adult strategy education that has 5As (assessment, advise, agree, assist and arrange), was presented in five 45-minute sessions (31). Other educational methods like lectures, questions and answers, and problem solving were also used. Information and educational materials like CDs, booklets, and pamphlets were also distributed among the participants. No educational sessions were conducted for the control group. The questionnaires were filled by the two groups 3 months after the intervention and then after the data were compared with the previous data. Similar educational sessions were conducted for the control group after the end of data collection. 

### 3.4. Ethical Consideration

After receiving the necessary authorizations, oral and written consent was obtained from the participants. They were assured that the data would remain confidential and used for the research purposes only. The participants were also given an unconditional and absolute right of withdrawal at any time.

### 3.5. Data Analysis

The normal assumption was checked by Normality Test in SPSS. The HPLB was not normal, but the histogram was normal. Descriptive statistics including distribution, mean, and standard deviation and analytical statistics including paired t-test were performed to compare the main variables before and after the intervention in the intervention and the control groups. Also the homogeneity of demographic variables was examined by Chi-square test in the two intervention and control groups before the intervention. The data were analyzed using SPSS version 20 and the statistical significance level was 0.05.

## 4. Results

The mean age of the participants in the current study was 65.9 ± 3.6 (range 60-73). Most of the subjects in the two groups under study were married (intervention = 63.8 %, control group = 50.9%), had primary level education (intervention = 40.1 %, control group = 54.7%), owned a house (intervention = 54.7 %, control group = 51.3%), had a stable financial status (intervention = 83.6%, control group = 82.8%), and had health insurance coverage (intervention = 88.8%, control group = 87.5%). Most of the subjects were living with their spouse and children (intervention = 76.3%, control group = 79.3%). Regarding the question as how do you assess your current health? (Perceived health status) most of them answered good (intervention = 62.1%, control group = 64.7%) (Table 1).

In this study, chi-square test results, before intervention, showed no statistically significant difference between intervention and control groups regarding the demographic variables including age, gender (male, female), health insurance status (Yes, No) and Perceived health status (good, moderate and poor). In other words, the distribution of those variables was equal before intervention between the intervention and control groups (P > 0.05). 

Paired T-test results showed statistically significant differences between the mean total score of HPBs and its subscales, before and after the intervention in the intervention group (Table 2). While no significant difference in the mean total score of HPBs and 6 subscales were found in the control group before and after the intervention (Table 2). Comparison of awareness of aging facts showed a significant difference before and after the intervention (P ≤ 0.001) in the intervention group, but not in the control group (Table 3). A positive significant correlation was also found between awareness of aging facts and HPBs (P ≤ 0.001).

**Table 1.** Demographic Characteristics of the Elders in the Intervention and the Control Groups (n = 232) ^a^

Characteristics	Intervention Group	Control Group
**Age**		
≤65	122 (52.6)	117 (50.4)
≥65	110 (47.4)	115 (49.6)
**Sex**		
Male	116 (50)	113 (48.7)
Female	116 (50)	119 (51.3)
**Education level**		
primary	93 (40.1)	127 (54.7)
Secondary	58 (25)	71 (30.6)
Higher	81 (34.9)	34 (14.7)
**Marital status**		
Married	148 (63.8)	118 (50.9)
Single	16 (6.9)	14 (6.0)
Widowed/Divorced	68 (29.3)	100 (43.1)
**Housing status**		
Owner	127 (54.7)	119 (51.3)
Rented	105 (45.3)	113 (48.7)
**Financial**		
Stable	194 (83.6)	192 (82.8)
Unstable	38 (16.4)	40 (17.2)
**History of chronic diseases**		
Yes	108 (46.6)	165 (71.1)
No	124 (53.4)	67 (28.9)
**Health insurance coverage**		
Yes	206 (88.8)	203 (87.5)
No	26 (11.2)	29 (12.5)
**Living structure **		
Family	177 (76.3)	148 (79.3)
Relative	16 (6.9)	29 (12.5)
Alone	39 (16.8)	19 (8.2)
**Perceived health status**		
Good	144 (62.1)	150 (64.7)
Moderate	65 (28)	58 (25)
bad	23 (9.9)	24 (10.3)

^a^ Data are presented as No. (%).

**Table 2.** Average Responses for Health Promoting Behaviors Variable Before and After the Educational Program (n = 232) ^a^

Variables	Before Intervention	After Intervention	P Value
**Health promoting behaviors(Total)**			
Intervention	132.2 ±19.7	147.7 ± 10	< 0.001
Control	134.3 ±8.9	132.9 ± 8.1	0.28
**Physical activity**			
Intervention	17 ± 4.7	21.3 ± 3	< 0.001
Control	19.3 ± 2.6	19.1 ± 2.8	0.64
**Nutrition**			
Intervention	23.9 ±4.6	26.3 ±4.5	< 0.001
Control	23 ±2.6	22.7 ±2.7	0.23
**Stress management**			
Intervention	20.11±3.51	22.4 ±2.3	< 0.001
Control	20.7 ± 2.4	20.5 ± 2.6	0.18
**Spiritual growth**			
Intervention	23.7 ± 4.8	25.9 ± 2.6	< 0.001
Control	23.69±3.5	23.5 ± 2.2	0.83
**Interpersonal relations**			
Intervention	24.3 ± 4.3	26.2 ± 2.9	< 0.001
Control	24 ± 2.6	23.8 ± 2.4	0.37
**Health responsibility**			
Intervention	23 ±4.7	25.5±3.1	< 0.001
Control	23.4 ±2.3	23.3 ± 2.4	0.09

^a^ Data are presented as mean ± SD.

**Table 3.** Average Responses for Facts on Aging variable Before and After the Educational Program (n = 232) ^a^

Variables	Before Intervention	After Intervention	P Value
**For facts on aging**			
Intervention	62.2 ± 4.8	72.7 ± 3.1	< 0.001
Control	61.4 ± 4.2	61 ± 4.9	0.11

^a^ Data are presented as Mean ± SD.

## 5. Discussion

Successful aging has a direct relationship with HPBs. Evidence shows that the elderly will have a better lifestyle when they observe HPBs as a routine correctly (27, 32). The present study aimed to evaluate the effect of an educational program based on the successful aging approach on HPBs in the elderly.

The mean total score of health-promoting behaviors showed a statistically significant difference between the control and the intervention groups, showing a significant increase after the intervention, indicating the effectiveness of such educational programs. The current study results are consistent with those of the studies by Heidari and Nasrabadi et al. (33, 34). Such behaviors would result in an improvement in the elderly people's lifestyle. In addition to the total score of HPBs, the aging educational programs also affected its subscales; after the intervention in the intervention group, the mean scores of different subscales of HPBs including physical activity, health responsibility, spiritual growth, nutrition, interpersonal relations, and stress management increased while no changes were observed in the control group showing the positive effect of educational programs on the subscales of HPBs. The results are in accordance with the findings of the study by of Robinson-Whelen et al. which aimed to empower the elderly women (35). In the mentioned study, total HPBs and 5 subscales in the intervention group increased significantly, showing that the program promoted healthy behaviors and physical health (35). Regarding the subscales of HPBs, the role of physical activity as an independent risk factor has been already proven in the lifestyle studies (22, 36). In the present study, the mean score of physical activity showed a statistically significant difference between the intervention and control groups after the intervention. Abedi et al. reported an increase in the physical activity in women with low physical activity levels after the intervention (37). Being active is the biggest challenge for the elderly. Therefore, to increase their physical activity, one should focus on different types of physical activities to lead them from being inactive to being more physically active. Of course, Bouchard and Ferrari also emphasized the necessity of focusing on educational interventions on physical activity and a healthy nutrition to have a healthy elderly population (38, 39). Nutrition was also studied as one of the subscales of HPBs and changes were observed in the nutritional habits of the elderly after the intervention as healthy eating behaviors significantly increased after the intervention in the intervention group, but not in the control group. The current study results are consistent with those of the similar studies (40, 41) which reported that education can cause significant changes in adopting HPBs related to nutrition. In this study, after implementing the educational program which aimed to identify the utilization of the stress control methods, the mean score of stress management changed significantly in the intervention group and educating the relaxation programs and techniques increased the adoption of coping strategies for the management of stressful situations while no changes were observed in the control group in this regard. The current study results are consistent with those of the studies (42-44) which reported that healthy lifestyle programs that include relaxation methods may ensure mental and physical health and decrease mental stress and its physical symptoms. In fact, relaxation techniques like jogging and anger management in line with the programs on successful aging can help to control stress and promote the elderly people’s mental health.

The mean score of mental and spiritual development significantly increased in the intervention group but not the control group, which is consistent with other studies (8, 11, 45). In a study by Ng et al. spirituality was one of the determining factors of successful aging in the Chinese elderly. However, it should be also mentioned that persuasive strategies and motivational approaches are necessary for the elderly to adopt an active lifestyle (46), since the mental health is desirable, the elderly are more motivated to perform HPBs. 

 It was also observed that the educational program significantly increased the mean score of interpersonal relationships only in the intervention group. Other studies have reported similar results and confirmed the positive significant relationship between social support and HPBs (22, 47). The elderly experience more social isolation and feel lonelier in comparison with other life stages. The social isolation results from retirement and lack of relationship with co-workers and friends in addition to several deaths (of wife, children, friends, family, co-workers, and etc.), and creates a sense of loneliness and depression in the elderly. Therefore, it is fundamental to pay attention to the interpersonal relationships and their continuity. Several studies have mentioned that loneliness intensifies depression and is a risk factor in elder abuse (48, 49). The elderly people’s participation in social activities appropriate with their mental and physical conditions promotes a sense of usefulness and releases them from isolation, loneliness, and depressive feelings. For this reason, in researches on the elderly, social support from family, friends, and acquaintances have always been in the center of the focus as an important factor (26, 50). 

After the intervention in the present study, the mean score of responsibility significantly increased only in the intervention group in comparison with the pre-intervention results, which is consistent with the results of other studies (5, 36, 51). Health responsibility is especially important in the elderly and ignoring it can result in irreversible damages and even early death in the elderly population. It is worth mentioning that educational programs are successful only if the elderly people’s knowledge and awareness of the facts and problems of the old age and the related issues are considered; this is why the elderly individuals’ knowledge was measured and its promotion was considered in the present research.

After the educational program, the mean score of the knowledge of aging facts significantly increased in the intervention group, but not in the control group, which is consistent with the previous results (34). In line with other studies (52), a positive significant correlation was also found between knowledge and HPBs. In fact, when the knowledge of the elderly on their physical, mental, and nutritional needs increases, they can adapt themselves to the aging better. Aging is one of the inevitable life stages. Aging cannot be prevented, but it can be postponed or its problems can be decreased through focusing on successful aging programs to transform it to an enjoyable stage.

Many of the old age problems result from unhealthy lifestyles at this stage of life. Focusing on successful aging and adopting HPBs can prevent and decrease aging problems which in turn decreases the financial burden and related costs. This is especially important for the policy and decision makers of the health system. Therefore, it is suggested that educational programs on HPBs start before the aging stage, in the middle age or even after childhood, to enhance their efficacy. Being RCT study is the strength point of this study.

Regarding the weaknesses of this study we can say that this was conducted on the elderly people who were admitted at Health Houses in 22 municipality diacritics in Tehran, and the elderly people in nursing homes were not studied. The designed intervention was elderly based which was one of the limitations of this study. Community-based interventions are suggested to be considered for planning other educational programs. The reliability of response bias could limit the validity of the results. Also the study sample was limited to those who could speak Persian.
